# Genome-Wide Analysis of YTH Domain Proteins in *Metasequoia glyptostroboides* and Functional Validation of MgYTH5 as an m^6^A Reader

**DOI:** 10.3390/plants15101497

**Published:** 2026-05-14

**Authors:** Bao Li, Xin Hu, Wenhui Guo, Huijuan Yin, Yuke Ma, Kongshu Ji, Qiong Yu

**Affiliations:** 1 State Key Laboratory of Tree Genetics and Breeding, Nanjing Forestry University, Nanjing 210037, China; libao131411@163.com (B.L.); hxneversaynever@163.com (X.H.); gwenhui1106@163.com (W.G.); yhuijuan2000@163.com (H.Y.); mayuke2001@163.com (Y.M.); ksji@njfu.edu.cn (K.J.); 2 Co-Innovation Center for Sustainable Forestry in Southern China, Nanjing Forestry University, Nanjing 210037, China; 3 Beijing National Laboratory for Molecular Sciences, Beijing 100190, China

**Keywords:** YTH domain, *Metasequoia glyptostroboides*, stress and gene expression analysis

## Abstract

*N*^6^-methyladenosine (m^6^A) is an important epigenetic modification of eukaryotic RNA, playing a significant role in various biological processes. *Metasequoia glyptostroboides* (*M. glyptostroboides*) is an ancient tree species in China, with a long history and excellent genetic characteristics. In this study, we identified six *MgYTH* genes in the genome of *M. glyptostroboides*, elucidating their phylogenetic relationships, conserved domains, gene structures, conserved motifs, chromosome locations, and prediction of LLPS. The analysis of the cis-regulatory elements in the promoter region suggested that *MgYTH* genes are associated with drought and the ABA-responsive expression patterns signaling pathway, which was further supported by expression pattern analysis. In addition, to directly evaluate the m^6^A binding ability of MgYTH proteins, we selected MgYTH5 as the representative for homology modeling analysis and electrophoretic mobility shift assay (EMSA). The results demonstrated that MgYTH5 has the ability to bind m^6^A in vitro, thereby providing biochemical evidence that MgYTH5 can bind m^6^A-modified RNA in vitro mRNAs. The subcellular localization results showed that MgYTH5 is located in the cytoplasm. These findings provide new insights into the epigenetic regulation mechanisms in gymnosperms and provide a resource for future functional studies in this species.

## 1. Introduction

m^6^A is the most widespread internal chemical modification in eukaryotic cell mRNA and plays critical roles in regulating post-transcriptional RNA processing and metabolism across diverse biological processes [1,2]. In mammals, m^6^A is dynamically regulated by methyltransferases (writers) that catalyze methylation, demethylases (erasers) that remove methyl groups, and m^6^A-binding proteins (readers) that recognize m^6^A-modified RNA and regulate gene expression [3,4]. Among them, m^6^A-binding proteins play significant roles by recognizing and exerting the corresponding functions of m^6^A methylation. Through specific interactions with m^6^A-modified transcripts, these readers influence multiple aspects of RNA metabolism, including mRNA splicing, degradation, translation initiation, and translation efficiency [5].

In plants, YTH domain-containing proteins are the major class of m^6^A readers and play important roles in plant growth and development [6]. In *Arabidopsis thaliana* (*A. thaliana*), several YTH domain proteins have been shown to play important roles in plant growth and development. For example, ECT2, ECT3, and ECT4 function redundantly to regulate leaf morphogenesis, and the delayed emergence of leaves in the *ect2*/*ect3*/*ect4* mutant is mainly caused by the slower growth rate of leaf primordia and primordium growth [7,8,9]. These proteins can directly interact with each other and enhance the binding efficiency of m^6^A-modified mRNAs, while also increasing transcript stability through the recruitment of poly(A)-binding proteins such as PAB2 and PAB4, thereby influencing plant developmental processes [10,11]. In addition, ECT2 has been reported to regulate the morphology of stipules by regulating transcriptional stability [12]. Another important m^6^A reader, CPSF30-L, which localizes to the liquid-like nucleus, participates in mRNA processing by regulating polyadenylation site selection. Loss of CPSF30-L leads to the extension of the 3′ non-coding region of the *SOC1* transcript, thereby accelerating transcript degradation and delaying flowering [13]. Recently, it was reported that the early-heading *DATE6* (*EHD6*) physically interacts with YTH07 through YTH07-mediated phase separation to promote accelerated flowering in rice [14].

Increasing evidence indicates that YTH domain proteins are involved in diverse physiological response processes in plants [15]. In tomato, knockout of the m^6^A reader SlYTHDF2 directly leads to an increase in endogenous ABA accumulation, which in turn accelerates leaf senescence [16], while mutation of SlYTH1 reduces seed germination rate [17]. In *Triticum aestivum*, the expression of the m^6^A binding protein TaETC9 is significantly induced under drought stress, and the *taect9* mutant showed enhanced sensitivity to drought conditions [18]. Similarly, in *Setaria italica*, mutation of the m^6^A reader SiYTH1 results in reduced stomatal closure and increased hydrogen peroxide accumulation during drought stress, ultimately affecting plant drought resistance [19]. In *Camellia chekiangoleosa*, the *CchYTH* genes are differentially expressed among tissues and are responsive to drought stress [20]. In apples, the YTH domain protein MhYTP2 has been studied and identified as a m^6^A reader, where MhYTP2 has both m^6^A-dependent and m^6^A-independent functions; overexpression of MhYTP2 reduces resistance to powdery mildew while enhancing tolerance to nitrogen deficiency [21,22]. Moreover, ECT8 and ECT12 have been proven to be able to regulate the stability of m^6^A-modified transcripts, thereby enhancing the environmental stress tolerance in *A. thaliana* [23,24]. ECT8 also participates in the negative feedback regulation of ABA signaling [25], whereas ECT1 has been shown to suppress salicylic acid-mediated stress responses [26]. Collectively, these studies demonstrate that YTH domain-containing proteins play crucial roles in mediating m^6^A-dependent regulation of plant growth, development, and stress responses. Therefore, systematic identification and functional characterization of YTH proteins are essential for further understanding the molecular mechanisms of m^6^A-mediated epigenetic regulation in plants.

As an important economic tree, *M. glyptostroboides* possesses multiple values in industrial production, economic development, medicinal utilization, scientific research and ecological environment construction. However, no study has been reported on the m^6^A readers, particularly YTH domain-containing RNA binding proteins in *M. glyptostroboides*. In this study, we analyzed chromosomal localization, phylogenetic trees and other features and identified six *MgYTH* genes in total. We further investigated their expression patterns and responses to various stress treatments. These results provide insights into the potential roles of *MgYTH* genes in the growth and development of *M. glyptostroboides*, offering a new perspective for the epigenetic study of this important tree species.

## 2. Results

### 2.1. Genome-Wide Identification and Basic Physicochemical Property Analysis of the MgYTH Genes in M. glyptostroboides

A genome-wide analysis of *M. glyptostroboides* identified six *MgYTH* genes, which were named *MgYTH1*-*MgYTH6* according to their chromosomal positions. The MgYTH proteins range from 236 to 925 amino acids in length, with an average of 608.1 amino acids. Their predicted molecular weights range from 24.47 kDa to 102.72 kDa, with an average of 67.61 kDa. Their isoelectric points (pI) vary between 5.58 and 9.91, with an average value of 7.19. Approximately 50% of MgYTH proteins are predicted to be stable, while the others (MgYTH1, MgYTH3, and MgYTH6) are predicted to be unstable, with instability index values exceeding 40. Subcellular localization predictions indicate that only MgYTH6 protein is nuclear, whereas the remaining five are cytoplasmic (Table 1).

**Figure 1:**
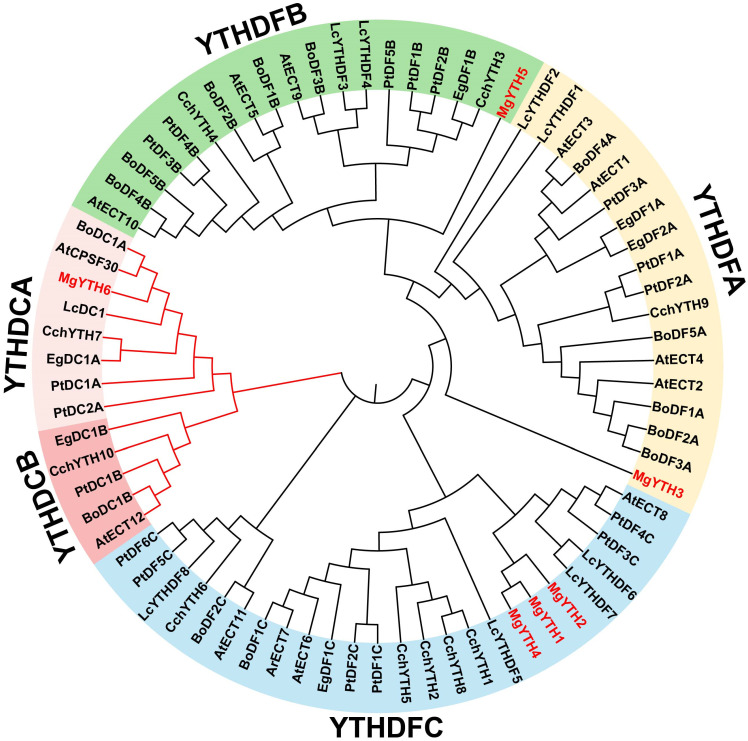
Phylogenetic relationship of the *MgYTH* gene family; different subfamilies are marked by different colored branches.

Based on studies of YTH proteins in other plants, the MgYTH family in *M. glyptostroboides* was classified into two groups: DC and DF. The DF group consists of 5 members (MgYTH1, MgYTH2, MgYTH3, MgYTH4, MgYTH5), whereas the DC group has 1 member (MgYTH6). To investigate evolutionary relationships, YTH domain-containing proteins from five plant species, together with the MgYTH proteins, were used to construct a phylogenetic tree, which was further divided into five subfamilies (see Supplementary Table S1). Among these, MgYTH6 is the sole member of the YTHDCA subfamily, MgYTH5 belongs to YTHDFB, and the YTHDFC subfamily contains three members (MgYTH1, MgYTH2, MgYTH4). The YTHDFA subfamily comprises a single member (MgYTH3). No members were identified in the YTHDCB subfamilies (Figure 1).

### 2.2. Analysis of Gene Structure, Conserved Domains and Motifs of MgYTH Genes in M. glyptostroboides

**Figure 2:**
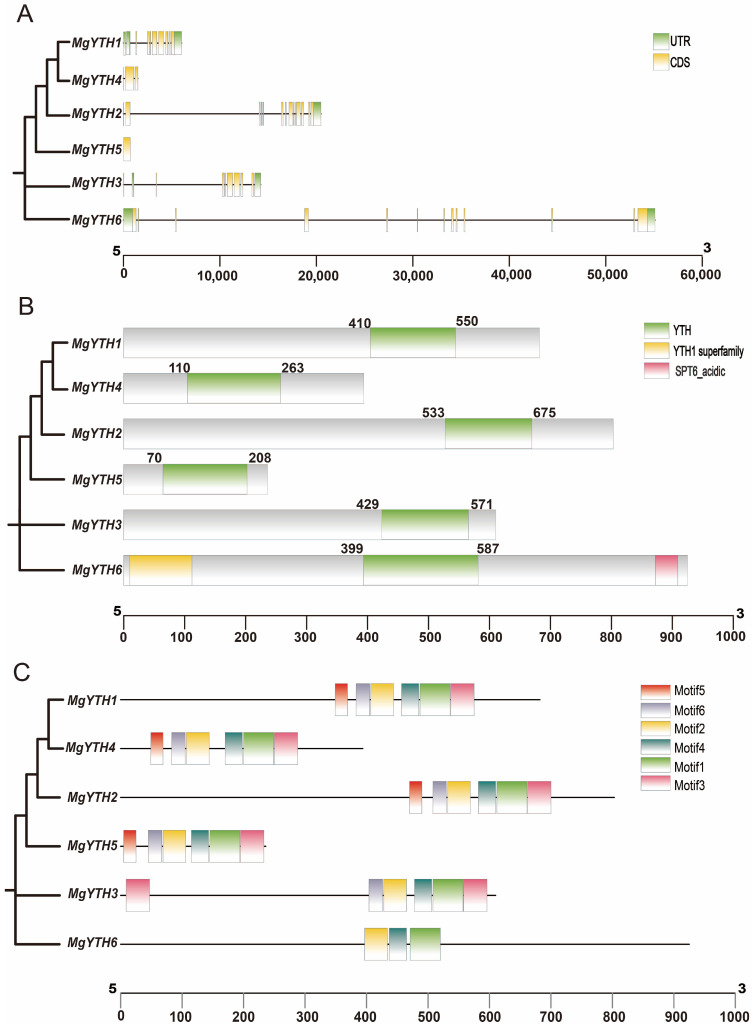
Structural and motif analysis of *MgYTH* genes and proteins in *M. glyptostroboides*. (**A**) Exon/intron organizations of *MgYTH* genes. Green boxes represent exons, and black lines represent introns. (**B**) The conserved YTH domain in MgYTH proteins is marked in green, and the numbers indicate the domain range. (**C**) Distributions of conserved motifs in MgYTH proteins. Six putative motifs are indicated in different colored boxes.

Exon–intron structure analysis of the *MgYTH* genes, combined with their evolutionary classification, revealed that all *MgYTH* genes contain introns, ranging from 1 to 13 per gene. The number of exons and UTRs varies significantly among all *MgYTH* genes, indicating the putative functional differences among these MgYTH proteins (Figure 2A). Conserved domain analysis showed that each MgYTH protein contains a typical functional YTH domain (Figure 2B), whereas MgYTH6 additionally contains an N-terminal SPT6 domain (Figure 2B). Motif analysis identified six conversed motifs across the MgYTH proteins. Motifs 1, 2, and 4 are present in all proteins, motif 6 is specific to the DF subfamily, and motif 5 is unique to the DC subfamily (Figure 2C).

### 2.3. Chromosome Localization of the MgYTH Genes in M. glyptostroboides

**Figure 3:**
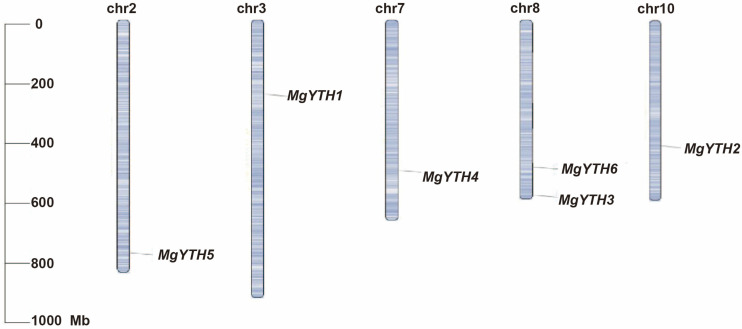
Chromosome localization and distribution of 6 *MgYTH* genes. Chromosome size is represented by relative length (millions of base pairs, Mb).

The *M. glyptostroboides* has 11 pairs of chromosomes, and 6 members of the *MgYTH* genes are unevenly distributed across 5 chromosomes (Figure 3). Except for chromosome 8, each chromosome contains only one *MgYTH* gene. No tandemly duplicated *MgYTH* genes were observed on any chromosome, and there was no positive correlation between chromosome length and the number of *MgYTH* genes. The uneven chromosomal distribution suggests potential diversification during evolution of the *MgYTH* gene family.

### 2.4. Prediction of LLPS in MgYTH Genes

**Figure 4:**
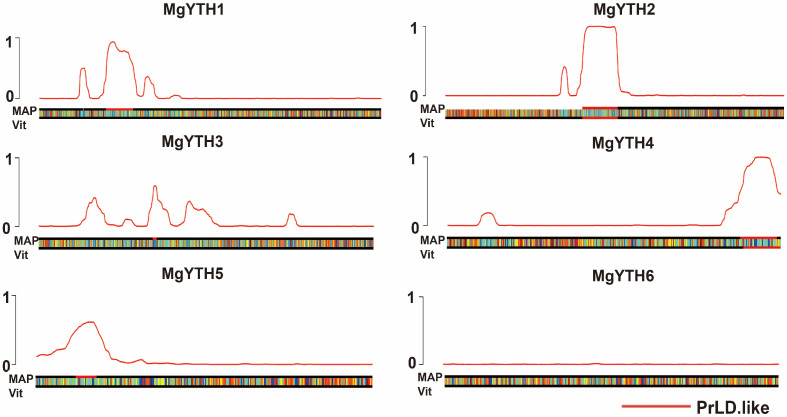
Prediction analysis of PrLDs in MgYTH proteins. The red outline represents the aggregating domains identified through calculation, and the region above the baseline threshold (black line) indicates the tendency of structural disorder, which is related to potential liquid–liquid phase separation.

Human m^6^A readers YTHDF1/3 undergo cellular LLPS, which is enhanced by multiple m^6^A modifications. To explore whether MgYTH proteins exhibit similar liquid-like properties, we analyzed the presence of prion-like domains (PrLDs). In our analysis, we found that MgYTH1, MgYTH2, MgYTH3, MgYTH4, and MgYTH5 were predicted to possess 1 or 2 highly disordered PrLD domains, while MgYTH6 lacked such PrLD region (Figure 4). This indicates that most MgYTH proteins may undergo phase separation in a manner similar to the human YTHDF1-YTHDF3 proteins, further supporting their potential liquid-like properties.

### 2.5. Comparative Analysis of Three-Dimensional Structures and Sequences of MgYTH Proteins

**Figure 5:**
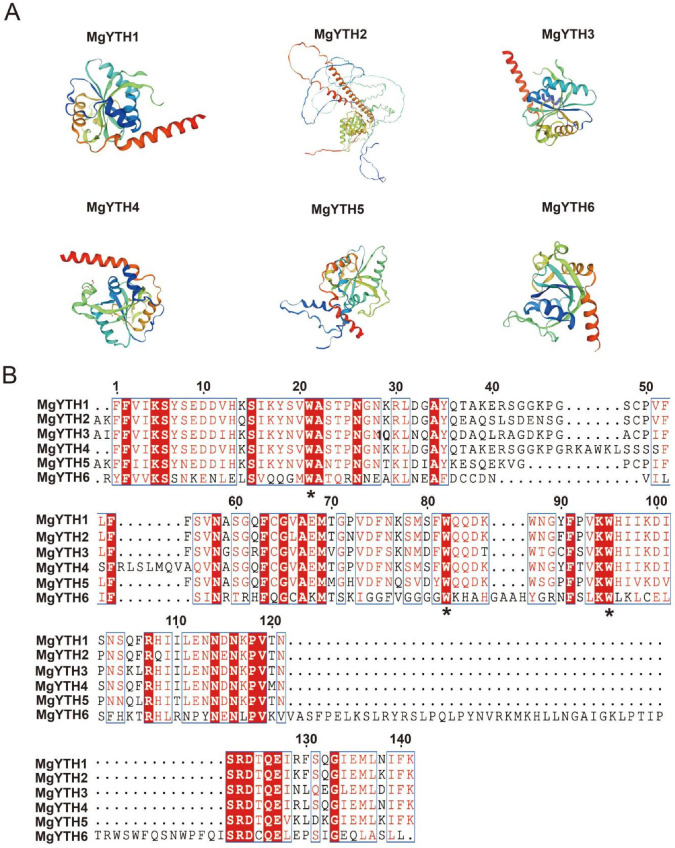
Analysis of the structural and sequence characteristics of the MgYTH proteins. (**A**) Three-dimensional protein structures of the YTH domains in MgYTH proteins. (**B**) Multiple sequence alignment of the YTH domains in the MgYTH protein family. The black asterisks indicate the amino acid residues that form the aromatic cage responsible for binding to m^6^A.

The three-dimensional structures of MgYTH proteins were predicted based on their amino acid sequences. The results showed that most of the MgYTH proteins were predicted to have similar three-dimensional structures, with greater structural similarity observed within subfamilies than between subfamilies. In general, these YTH domains usually adopt a specific mixed α-helix-β-sheet folding, where the β-sheet is arranged in a β-tube structure and is surrounded by α-helices (Figure 5A). The multiple sequence alignments of the MgYTH proteins showed that many functional sites were highly conserved including the characteristic aromatic cages. Notably, all members of the DF subfamily possess aromatic cages composed of three tryptophan residues (WWW) (Figure 5B).

### 2.6. Analysis of Cis-Regulatory Elements in the Promoter MgYTH Gene Family

**Figure 6:**
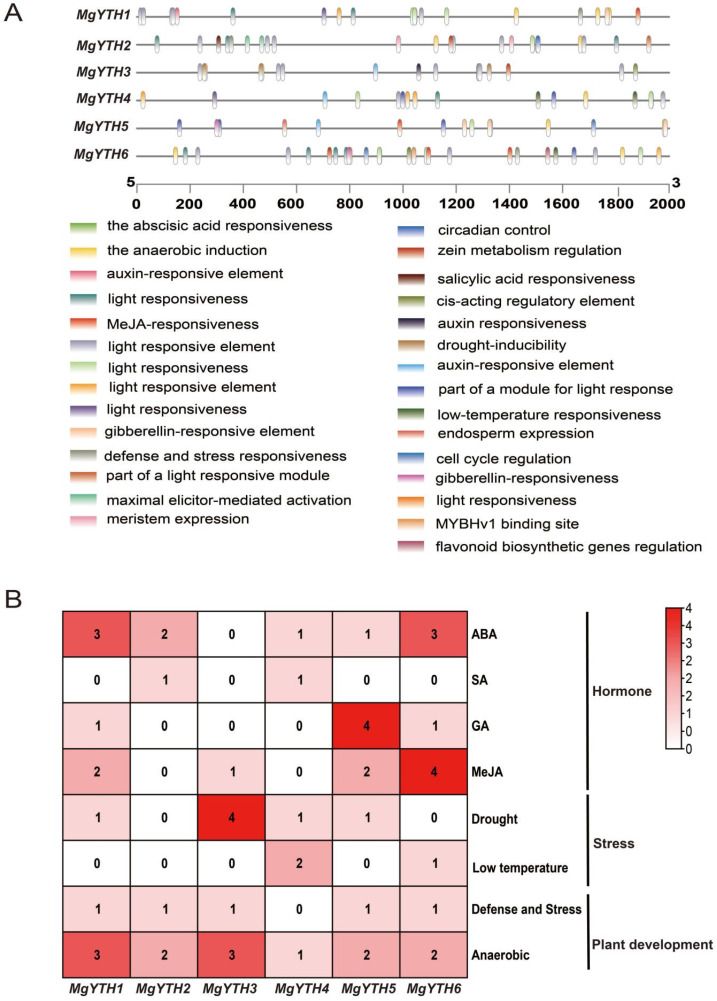
Analysis of cis-regulatory elements in the promoters of *MgYTH* genes. (**A**) Distribution of predicted cis-regulatory elements in the 2000 bp upstream promoter regions of *MgYTH* genes. Different colored boxes represent different types of cis-regulatory elements. (**B**) Number of major cis-regulatory elements of six *MgYTH* genes. The blank boxes indicate the absence of a specific cis-element, while different colors represent different element counts.

To further investigate the potential regulatory mechanisms of the *MgYTH* gene, we used the online tool PlantCare (https://bioinformatics.psb.ugent.be/webtools/plantcare/html/) to analyze the cis-regulatory elements present in their promoter regions. A total of 29 different types of cis-regulatory elements were identified, which may be involved in various growth and developmental processes (Figure 6A). We further statistically analyzed the number of several major cis-regulatory elements related to MeJA, ABA, drought, low temperature, GA, SA, defense mechanisms, and circadian rhythm control (Figure 6B). Among them, ABA and drought-responsive elements were the most abundant, which to a certain extent implies that *MgYTH* genes may be involved in plant stress responses and hormone signaling regulation.

### 2.7. Expression Pattern Analysis of the MgYTH Gene Family

**Figure 7:**
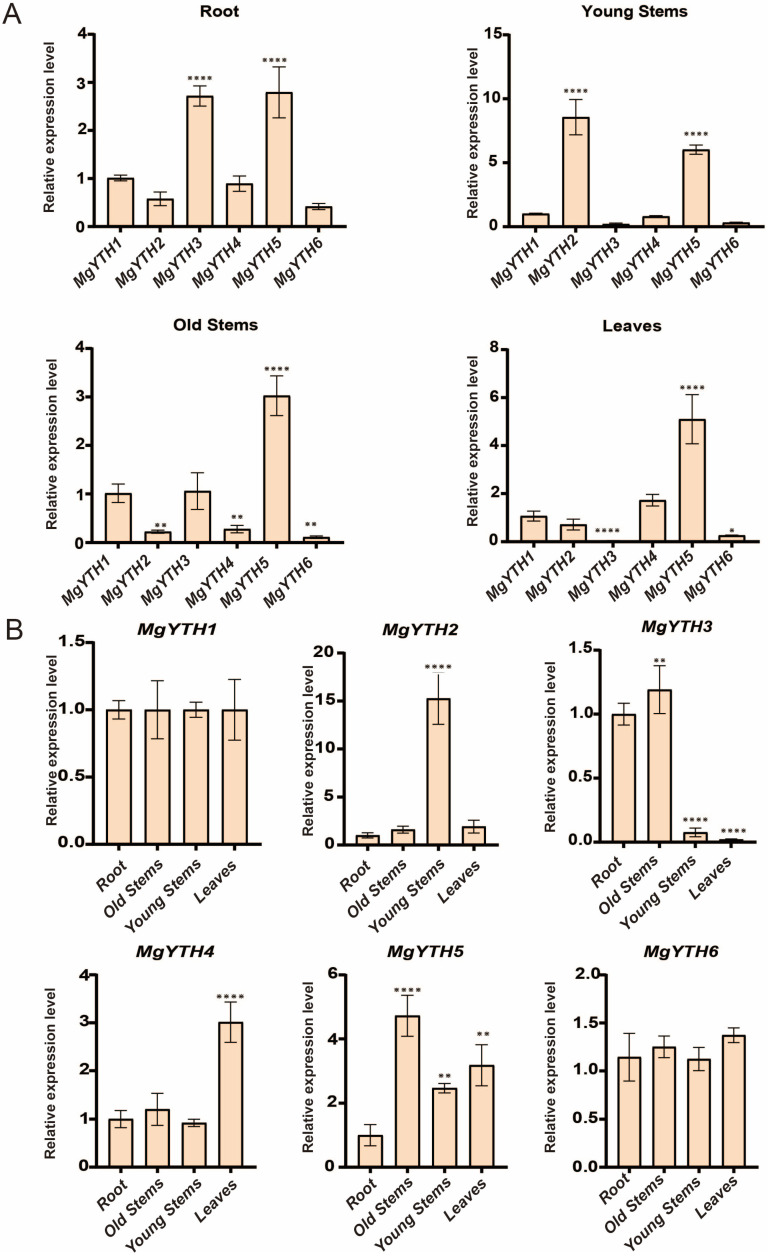
Tissue-specific analysis of six *MgYTH* genes. (**A**) Expression patterns of six *MgYTH* genes in different tissues. (**B**) Expression of six *MgYTH* genes in root, old stems, young stems, and leaves. *: *p* < 0.05, **: *p* < 0.01, ****: *p* < 0.0001, Relative expression levels were normalized using *ACT2* as the reference gene. Data represent mean ± SE (*n* = 3 biological replicates).

In order to study the physiological role of the *MgYTH* gene in growth and development, specific quantitative primers were designed (see Supplementary Table S2) and the tissue-specific expression patterns of *MgYTH* genes were analyzed in four different tissues (root, old stems, young stems, and leaves). The results showed that all *MgYTH* genes were expressed in the tested tissues but exhibited distinct expression patterns (Figure 7A). *MgYTH3* and *MgYTH5* were highly expressed in the root, whereas *MgYTH2* and *MgYTH5* were highly expressed in the young stems. *MgYTH5* was also highly expressed in leaves and old stems. In contrast, *MgYTH3* showed relatively low expression levels in young stems and leaves. Further analysis revealed that the expression levels of *MgYTH2*, *MgYTH3*, *MgYTH4*, and *MgYTH5* genes were significantly different in different tissues, while *MgYTH1* and *MgYTH6* showed no significant differences across the four tissues (Figure 7B).

### 2.8. Differential Expression Analysis of MgYTH Genes Under ABA and Drought Stresses

**Figure 8:**
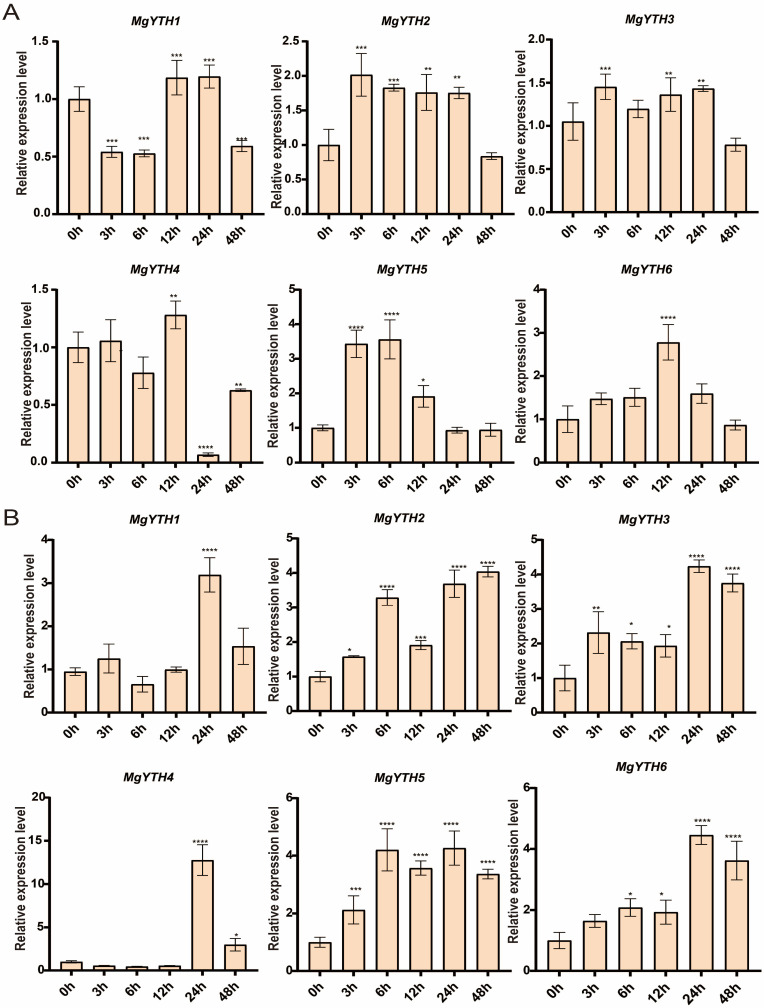
Expression profiles of *MgYTH* genes under ABA and drought stress treatments. (**A**) Expression pattern of the six *MgYTH* genes in leaves under ABA treatment. (**B**) Expression pattern of the six *MgYTH* genes in leaves under drought stress. Data represent mean ± SE (*n* = 3 biological replicates). The asterisks positioned above the bars signify statistically significant differences as determined by one-way ANOVA. * *p* < 0.05, ** *p* < 0.01, *** *p* < 0.001, **** *p* < 0.0001.

Promoter analysis revealed that *MgYTH* genes contain numerous cis-regulatory elements associated with stress responses. To further investigate their potential roles in stress adaptation, the expression patterns of *MgYTH* genes under ABA and drought treatments were analyzed using *M. glyptostroboides* seedlings. The results showed that after ABA treatment, *MgYTH1*, *MgYTH2*, and *MgYTH5* showed significant changes in expression following ABA treatment stimulation, exhibiting significant expression changes at different time points (Figure 8A). *MgYTH3* showed relatively high expression at 3 h, *MgYTH6* reached its highest expression level at 12 h, and *MgYTH4* showed a significant decrease in expression at 24 h. The remaining genes showed no significant differences. Under PEG-simulated drought treatment, most *MgYTH* genes were involved in the regulation of drought stress (Figure 8B). *MgYTH1*, *MgYTH3*, *MgYTH4*, and *MgYTH6* reached their highest expression level at 24 h and subsequently showed a downward trend. In contrast, *MgYTH2* and *MgYTH5* showed an increase first at 6 h, then decreased at 12 h, and continued to increase again at 24 h and 48 h.

### 2.9. Subcellular Localization Analysis

**Figure 9:**
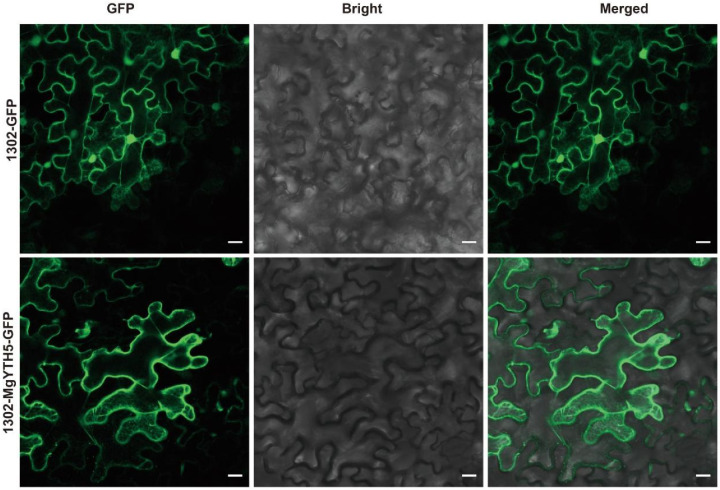
Subcellular localization of MgYTH5 protein in transiently transformed *N. benthamiana* leaf cells. The scale indicates 20 μm.

To investigate the subcellular localization of the *MgYTH* gene family, we conducted transient expression experiment in *Nicotiana benthamiana* leaves to determine the subcellular localization of MgYTH5 proteins. Since the *MgYTH5* gene showed high expression levels in root, stems, and leaves, MgYTH5 was selected as a representative for further analysis. The results showed that MgYTH5-GFP fluorescence was exclusively observed in the cytoplasm (Figure 9), which confirmed that this protein was located in the cytoplasm as predicted.

### 2.10. MgYTH5 Protein Specifically Bind to m^6^A-Modified RNA In Vitro

**Figure 10:**
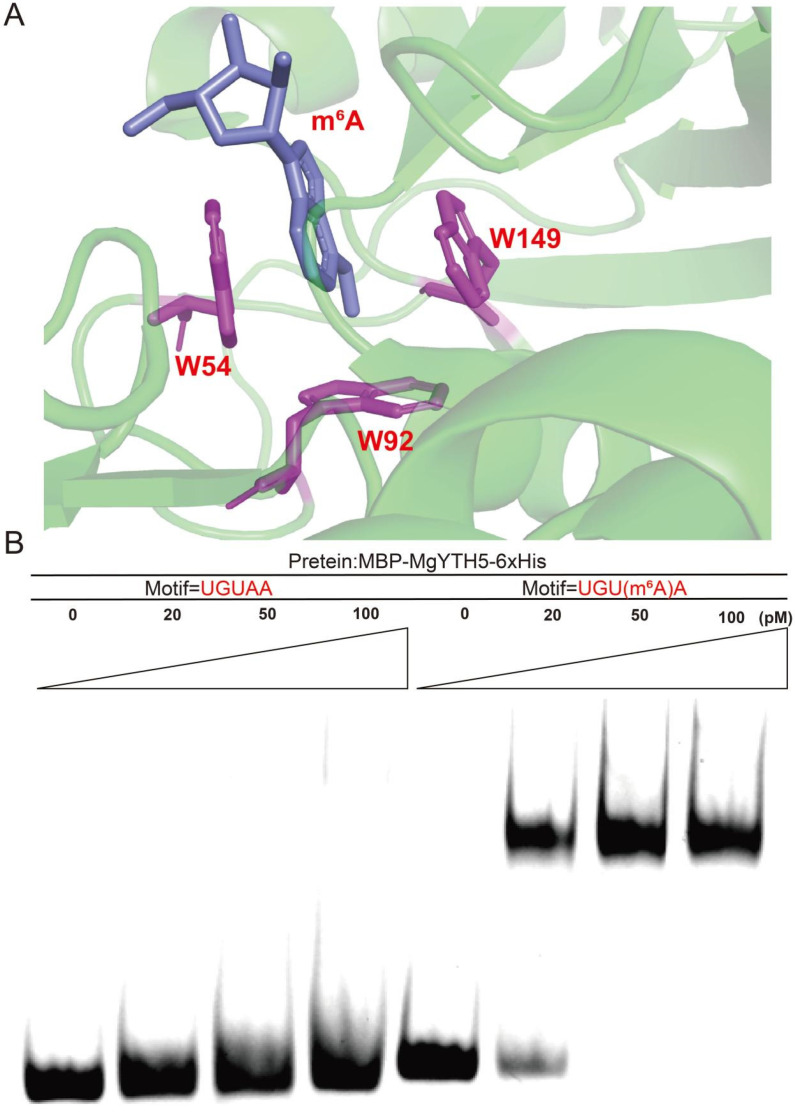
Structural basis and biochemical validation of m^6^A recognition by MgYTH5. (**A**) Three-dimensional structure of MgYTH5 bound to m^6^A modified RNA, in which an aromatic cage formed by three tryptophan residues mediates m^6^A recognition. (**B**) EMSA showing the binding of MgYTH5-6×His to methylated and unmethylated RNA probes. FAM-labeled RNA probes (4 nmol) were incubated with increasing concentrations of MgYTH5-6×His protein (0–100 pM). Fluorescently labeled RNA oligonucleotides of conserved motifs from model plants were used: 5′-UCUUUGUXAGACUGUACUCUUUA-3′, where X represents A or m^6^A.

Phylogenetic analysis indicated that MgYTH5 belongs to the YTHDF branch and has been confirmed as a close relative homolog that is extremely similar to the *A. thaliana* m^6^A reader AtECT2. Homology modeling showed that there was a conserved m^6^A binding pocket in MgYTH5, where three tryptophan residues form a typical aromatic cage capable of accommodating methylated adenine (Figure 10A). To verify its m^6^A binding activity, MgYTH5 was selected as a representative member of the *MgYTH* gene family for EMSA. The recombinant MgYTH5 with a C-terminal 6×His tag was heterologously expressed in *Escherichia coli* BL21 (DE3) and purified by Ni-NTA affinity chromatography (see Supplementary Figure S1). The purified protein was incubated with FAM-labeled RNA probes containing either unmodified A or m^6^A. MgYTH5 clearly bound to the m^6^A-modified RNA probe, while the binding to unmodified RNA was weaker or undetectable (Figure 10B). These results indicated that MgYTH5 shows preferential binding to m^6^A-modified RNA in vitro, confirming its function as an m^6^A reader protein.

## 3. Discussion

m^6^A has emerged as one of the most prevalent epitranscriptomic marks in eukaryotes and plays essential roles in RNA metabolism and gene regulation [27]. As key effectors of m^6^A signaling, YTH domain-containing proteins function as m^6^A readers that specifically recognize methylated RNA and mediate downstream regulatory processes, including RNA stability, translation, and alternative splicing [28]. Although YTH family proteins have been extensively studied in many plants, their characteristics and functions remain largely unknown in *M. glyptostroboides*. We conducted a thorough investigation to identify and characterize the *YTH gene* family within the species *M. glyptostroboides*. This comprehensive analysis yielded valuable insights into the structural characteristics of these genes, as well as their potential biological roles and functions. Our findings contribute to a deeper understanding of the YTH gene family, highlighting its importance in the context of evolutionary biology and gene function in this particular organism.

A total of six *MgYTH* genes were identified from the *M. glyptostroboides* genome and classified into two major subfamilies, YTHDF and YTHDC, based on phylogenetic analysis. The YTHDF subfamily contains five members (*MgYTH1-MgYTH5*), whereas the YTHDC subfamily includes only one member (*MgYTH6*). We found that the YTHDFB member is absent in *M. glyptostroboides*, which we speculate may be attributed to gene loss during the evolution of gymnosperms. Similar gene family compositions have been reported in other plant species, where YTHDF proteins generally represent the dominant group of m^6^A readers that function primarily in the cytoplasm, while YTHDC proteins often may be involved in nuclear RNA processing [29]. The uneven chromosomal distribution of *MgYTH* genes observed in this study may reflect evolutionary diversification mechanisms.

Protein structure analysis revealed that all MgYTH proteins contain a conserved YTH domain, which is responsible for recognizing m^6^A-modified RNA. Sequence alignment further demonstrated that the aromatic cage responsible for m^6^A recognition is highly conserved among MgYTH proteins and consists of three tryptophan residues. This structural feature is widely recognized as the core determinant for m^6^A recognition in YTH domain proteins across eukaryotes [30]. The conservation of this structural motif suggests that MgYTH proteins likely retain canonical m^6^A-binding functions similar to those reported in other plant species [31]. Interestingly, MgYTH6 contains an additional CCCH-type zinc finger motif, which may indicate functional divergence within the MgYTH family and suggests that this protein may be involved in distinct RNA regulatory processes.

Gene expression analysis revealed that *MgYTH* genes were widely expressed across different tissues, although their expression levels vary considerably. Most *MgYTH* genes exhibited relatively high expression in stems and leaves, indicating potential roles in actively growing tissues and metabolic processes. In contrast, relatively lower expression levels were observed in root, suggesting possible tissue-specific regulatory functions. Furthermore, promoter analysis revealed that the upstream regions of *MgYTH* genes contain multiple cis-regulatory elements associated with hormone signaling and stress responses. Consistent with this observation, several *MgYTH* genes exhibited dynamic transcriptional responses under ABA and drought stresses. These results suggest that *MgYTH* genes may be involved in plant stress-response pathways. Previous studies have demonstrated that m^6^A modification plays important roles in plant responses to abiotic stress by regulating RNA stability, translation efficiency, and stress-related gene expression [32]. Therefore, the stress-responsive expression patterns observed in this study imply that MgYTH proteins may contribute to stress-responsive regulatory pathways environmental adaptation in *M. glyptostroboides*.

Among the identified *MgYTH* members, *MgYTH5* attracted particular attention due to its relatively high expression levels across tissues. Subcellular localization analysis confirmed that MgYTH5 is predominantly localized in the cytoplasm, which is consistent with the typical localization pattern of YTHDF proteins that regulate mRNA stability and translation in the cytoplasm [33]. Furthermore, homology modeling revealed the presence of a conserved m^6^A-binding pocket in MgYTH5. Biochemical validation using EMSA demonstrated that MgYTH5 specifically binds to m^6^A-modified RNA in vitro. These results provide direct experimental evidence that MgYTH5 functions as a m^6^A reader protein.

Taken together, our findings not only revealed the evolutionary conservation and structural features of MgYTH proteins but also highlight their potential roles in plant development and stress responses. In particular, the identification and functional validation of MgYTH5 as an m^6^A-binding protein provide an important foundation for future studies aimed at elucidating the regulatory mechanisms of m^6^A-mediated RNA modification in *M. glyptostroboides*. Further investigations integrating genetic approaches, transcriptome-wide m^6^A mapping, and functional analyses will help to clarify the biological roles of MgYTH proteins in plant growth, development, and environmental adaptation.

## 4. Materials and Methods

### 4.1. M. glyptostroboides Materials and Growth Conditions

Young saplings of *M. glyptostroboides*, which were one year old, were cultivated on the campus of Nanjing Forestry University. These saplings were grown in a controlled growth medium maintained at a temperature of 25 °C, following a light cycle of sixteen hours of illumination and eight hours of darkness. This experiment was conducted in the National Key Laboratory of Tree Genetic and Breeding at the university, which provided the ideal conditions for studying the plants. For the purpose of spatial expression analysis, various plant parts were collected, including roots, old stems, young stems, and leaves. In conducting assays to assess the plants’ responses to abiotic stress, researchers utilized a 100 μM solution of abscisic acid (ABA) and a 20% solution of polyethylene glycol to irrigate the growth medium. These substances were selected to simulate stress conditions that the plants might encounter in their environment [34,35]. To thoroughly investigate the effects of these stressors, leaf samples from each treatment group were systematically collected at multiple time points: 0 h, 3 h, 6 h, 12 h, 24 h, and 48 h post-stress treatment. Importantly, samples taken at the 0 h mark from the control group, which experienced no stress, were used as a baseline for comparison. All experimental treatments were replicated three times biologically and in three technical replicates, ensuring robustness and reliability in the data collected.

### 4.2. Identification of the MgYTH Genes in the M. glyptostroboides

To identify the genes that encompass the YTH domain in *M*. *glyptostroboides*, the researchers began by downloading the relevant gene annotations and genomic files. These important resources were acquired from the NGDC (https://ngdc.cncb.ac.cn/), which provides comprehensive genomic information [36]. The Hidden Markov Model implemented in the HMMER3.0 program was subsequently utilized to conduct a search for the YTH521-B domain within the genome repository of *M. glyptostroboides*, as referenced in [37]. This search was performed with a stringent e-value threshold set at 1 × 10^−5^, ensuring high specificity in identifying relevant sequences. Following the identification of potential domains, various amino acid properties were analyzed using the ProtParam tool (https://web.expasy.org/protparam/). These properties included the molecular weight (MW), the instability index, hydrophilicity, and the isoelectric point (PI), all of which are critical for understanding the biochemical characteristics and potential functionality of the identified proteins [38]. The subcellular localization was predicted using the Cell-PLoc online tool (http://www.csbio.sjtu.edu.cn/bioinf/plant-multi/).

### 4.3. Phylogenetic Analysis and Chromosome Localization

In order to study the phylogenetic relationship among MgYTH proteins, the YTH protein sequences of *A. thaliana*, *Eucalyptus grandis*, *Populus tomentosa*, *Liriodendron chinense*, *C. chinensis* and *M. glyptostroboides* were collected and compared. Multiple sequence alignment of YTH protein sequences from *M. glyptostroboides* and other species were performed using MEGA 11. Based on the sequence alignment results generated via ClustalX (https://evomics.org/resources/software/bioinformatics-software/clustal-x/), a phylogenetic tree was constructed with the Maximum Likelihood (ML) algorithm. Bootstrap analysis was carried out with 1000 replicates to evaluate tree reliability. The final phylogenetic tree was further visualized and annotated using the online tool iTOL (https://itol.embl.de/) [39]. The protein amino acid sequences and corresponding gene identification numbers were deposited in the Supplementary Material. Information concerning the chromosomal localization of MgYTH family genes was extracted from the genomic GFF file of *M. glyptostroboides*. The chromosome location map of the *MgYTH* genes was generated using TBtools (v2.322).

### 4.4. Gene Structure, Protein Structure, Conserved Motif Analysis and Phase Separation Prediction

Information regarding genetic structure, encompassing exon–intron organization, was sourced from genome annotation files and was visualized with TBtools. The conserved motifs belonging to the MgYTH proteins underwent analysis via MEME (https://meme-suite.org/meme/) [40]. A maximum of six protein motifs were predicted, each ranging in length from 15 to 50 nucleotides. These MEME output files received further modifications through TBTools. The structural domains of the MgYTH proteins were examined using a database accessed on 31 May 2025. We utilized SWISS-MODEL (https://swissmodel.expasy.org/interactive) to predict the three-dimensional structural models for the YTH protein members of *M. glyptostroboides*. Multiple sequence alignments of the proteins were achieved with ESPript 3.0 (https://espript.ibcp.fr/ESPript/ESPript/) We employed PLAAC (http://plaac.wi.mit.edu/) [41] to forecast prion-like domains and disordered protein regions. The predictions utilized the default parameter settings. Visualizations of these proteins were conducted based on the findings from PLAAC and subsequently refined.

### 4.5. Analysis of the Cis-Regulatory Elements of the MgYTH Gene Promoters

For every gene investigated in this study, the promoter region was delineated as the genomic sequence extending 2000 base pairs upstream of its transcription start site. To identify cis-regulatory elements located within these promoter regions, the PlantCare (https://bioinformatics.psb.ugent.be/webtools/plantcare/html/) database was employed. Subsequently, the identified elements were visualized utilizing the software TBtools, enhancing the understanding of the regulatory landscape surrounding each gene’s promoter.

### 4.6. Investigation on Subcellular Positioning of MgYTH5 Protein

Primers *MgYTH5*-F and *MgYTH5*-R (see Supplementary Table S2) were used to amplify the coding sequences of *MgYTH5*. The sequences of *MgYTH5* were respectively cloned into the plant expression vector pCAMBIA1305, which was cut by XbaI and SalI enzymes and driven by the CaMV35S promoter between the C-terminal EGFP. The vector containing p35S: *MgYTH5*-EGFP was successfully introduced into *Agrobacterium* cells of the EHA105 strain and subsequently co-cultured with *p19*, which serves as an RNA silencing inhibitor, as referenced in [42]. Following this procedure, the *Agrobacterium* strain was then injected into the leaves of *N. benthamiana* to facilitate transient expression of the target protein. After allowing the plants to be cultured in darkness for a duration of three days, the GFP signal within the infiltration zone was visualized and captured using a confocal microscope set to an excitation wavelength of 488 nm.

### 4.7. Extraction of Total RNA and Expression Analysis via qRT-PCR of MgYTH Genes

Total RNA was extracted from the tissues of one-year-old *M. glyptostroboides* using RNA extraction kit (RC401, Vazyme Biotech, Nanjing, China) according to the manufacturer’s protocol. The concentration and purity of the total RNA were determined using Nano-Drop 2000 (Thermo Fisher Scientific, Waltham, MA, USA). Using 1 μg of total RNA as the template, first-strand cDNA synthesis was performed with the kit purchased from Shanghai YEASEN Biotechnology(Shanghai, China). The qPCR reaction was performed using the StepOne Plus real-time polymerase chain reaction system (Applied Biosystems, Foster City, CA, USA). The composition of the reaction mixture consisted of several key components essential for the experiment. Specifically, it included 1 µL of complementary DNA (cDNA) that had been diluted to a twenty-fold concentration. In addition to the cDNA, the mixture incorporated 0.4 µL of both the forward and reverse primers, each with a concentration of 10 µM. To facilitate the amplification process, 10 µL of SYBR Green Master Mix (Yeasen Biotechnology, Shanghai, China) was also added to the mixture. Finally, 8.2 µL of double-distilled water (ddH_2_O) was included to adjust the solution to a final volume of 20 µL, which is detailed in the primer sequence provided (see Supplementary Table S2). The amplification conditions were as follows: 2 min of pre-denaturation at 95 °C, 40 cycles, 10 cycles of denaturation at 95 °C, and 30 cycles of extension at 60 °C. *MgACT2* was used as a reference control gene. The data are shown as the mean ± SE, with three biological replications and three technical replicates in each experiment. The relative expression level of the gene was determined using the 2^−∆∆CT^ method [43].

### 4.8. Protein Homology Modeling and EMSA Experiment

We used the SWISS-MODEL (https://swissmodel.expasy.org/interactive) [44] to construct the model structures of the YTH structure-m^6^A RNA complex and MgYTH5, highlighting the amino acid residues interacting with m^6^A. To further explore and confirm the specific binding and recognition ability of MgYTH to m^6^A RNA, we chose MgYTH5 as an example. First, we constructed the pET28a-*MgYTH5*-MBP-6×His tag vector. Then, it was transformed into *Escherichia coli* (BL21(DE3)) and 0.5 Mol IPTG was added to the bacterial solution. The protein expression was induced at 18 °C, and the target protein was purified using the NiNTA-His tag purification column [45]. Then, EMSA experiments were conducted. The recombinant protein 6×His-MgYTH5 was diluted to a series of concentration ranges (from 0 to 80 pM) in the binding buffer, mixed with 1 μL of the protein in 10 μL of the binding buffer, and incubated on ice for 45 min. The entire RNA–protein mixture was loaded onto a Novex 16% TBE gel and electrophoresed at 4 °C [46]. The gel was visualized by ChemiDoc (Blue Nucleic Acid Gel tanon). The following fluorescently labeled RNA oligonucleotides of conserved motifs from model plants were used: 5′-FAM-UCUUUGUXAGACUUGUACUCUUA-3′ (X = A or m^6^A).

### 4.9. Statistical Analysis

All experimental data in this study were rigorously analyzed using the professional statistical software GraphPad Prism (version 9.5.0). Each experiment was performed with three independent biological replicates, and each biological replicate included three technical replicates. Prior to statistical analysis, the three technical replicates within each biological replicate were averaged, and the mean values of the biological replicates were used for subsequent statistical tests. Intergroup differences were assessed by one-way analysis of variance (one-way ANOVA), followed by Dunnett’s multiple comparisons test to compare each treatment group with the untreated control group. GraphPad Prism 9.5.0 uses the Brown–Forsythe test by default for homogeneity of variances during one-way ANOVA. All experimental data are presented as the mean ± standard error (SE). Statistical significance levels are indicated by asterisks: single asterisk, * *p* < 0.05; double asterisk, ** *p* < 0.01; triple asterisk, *** *p* < 0.001; quadruple asterisk, **** *p* < 0.0001. Furthermore, the gene expression levels measured in untreated samples served as the baseline control for all statistical analyses in this study.

## Figures and Tables

**Table 1:** Physicochemical properties and subcellular localization predictions of MgYTH proteins in *M. glyptostroboides*.

Gene Name	Gene ID	Number of Amino Acids	Molecular Weight	Theoretical pI	The Instability Index	Prediction of Subcellular Localization
*MgYTH1*	Mgl0004630.1	682	74.00	6.06	41.56	Cytoplasm
*MgYTH2*	Mgl0277390.1	803	89.50	6.50	36.57	Cytoplasm
*MgYTH3*	Mgl0091190.1	610	68.26	5.58	45.29	Cytoplasm
*MgYTH4*	Mgl0062540.1	394	43.71	9.15	32.04	Cytoplasm
*MgYTH5*	Mgl0093410.1	236	27.47	8.82	25.79	Cytoplasm
*MgYTH6*	Mgl0085950.1	925	102.72	7.77	54.76	Nucleus

## Data Availability

The original contributions presented in this study are included in the article/Supplementary Material. Further inquiries can be directed to the corresponding author.

## Supplementary Materials

The following supporting information can be downloaded at: https://www.mdpi.com/article/10.3390/plants15101497/s1, Figure S1: Protein purification. pET28a-MBP-MgYTH5-6×His were purified from *E. coli*; Table S1: Sequences in the evolutionary tree; Table S2: Primer sequences.
