# Supplementary material for: Genome-Wide Analysis of YTH Domain Proteins in Metasequoia glyptostroboides and Functional Validation of MgYTH5 as an m6A Reader
Source: Plants (Basel). 2026 May 14;15(10):1497. doi: 10.3390/plants15101497 (PMC13210855; doi:10.3390/plants15101497)
Supplement: Supplementary file 1 [file plants-15-01497-s001.zip › plants-4278434-supplementary.pdf]

Supplemental Figures

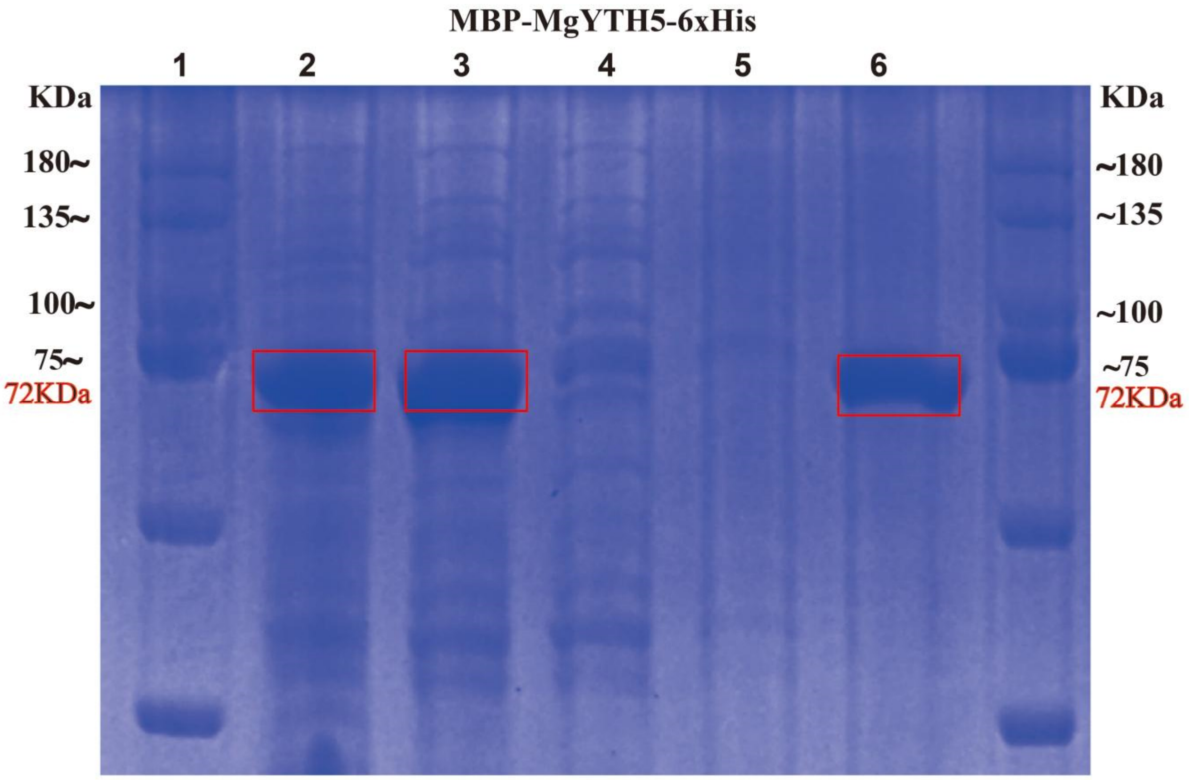

Figure S1. Protein purification. PET28a-MBP-MgYTH5-6×His were purified from *E. coli*. 1:Marker; 2: precipitate; 3: supernatant; 4: Flow-through; 5: 15 mM imidazole effluent; 6: the purified recombinant proteins.

Table S1. Sequences in the evolutionary tree.

Species name	Accession number	name code	sequnenceYTH motif
Arabidopsis thaliana	AT3G03950	ETC1	DVSAVDLQRYNGENFPESFVKAKFFVIKSYSEDDVHNCIKYGAWSSTPTGNKKLNAAYYEAKENSQECPVYLLFSVNASGQFVGLAEMVGPVDFNKTMEYWQQDKWIGCFPVKWHIIKDIPNSLLRHITLANNENKPVTNSRDTQEVNLEHGTKIIKIFKEYMSKTCILDDYKFYETRQKIIRDKKIKQKKQ
	At3g13460	ECT2	DNTCVVPDREQYNKEDFPVDYANAMFFIIKSYSEDDVHKSIKYNVWASTPNGNKKLAAAYQEAQQKAGGCPIFLFFSVNASGQFVGLAEMTGPVDFNTNVEYWQQDKWTGSFPLKWHIVKDVPNSLLKHITLENNENKPVTNSRDTQEVKLEQGLKIVKIFKEHSSKTCILDDFSFYEVRQKTILEKKAKQTQK
	AT5G61020	ECT3	SEDVSLLDPKDYNKIDFPETYTEAKFYVIKSYSEDDIHKSIKYSVWSSTPNGNKKLDASYNEAKQKSDGCPVFLLFSVNTSGQFVGLAEMVGPVDFNKTVEYWQQDKWIGCFPVKWHFVKDIPNSSLRHITLENNENKPVTNSRDTQEVKLEQGIKVIKIFKDHASKTCILDDFEFYENRQKIIQERKSKHLQI
	AT1G55500	ECT4	TVTCVLPDREECNRDDFPVEYKDAKFFIIKSYSEDDVHKSIKYNVWASTPNGNKKLDAAYQEAQQKSSGCPVFLFFSVNASGQFIGLAEMKGPVDFNKNIEYWQQDKWTGSFPLKWHILKDVPNSLLKHITLEYNENKPVTNSRDTQEVKLEQGLKVVKIFKEHNSKTCILDDFSFYEARQKTILEKKAKQQQS
	AT3G13060	ECT5	NKGSAKEHEESNNADFVTDYTNAKLFIIKSYSEDNVHKSIKYNVWASTPNGNKKLDAAYREAKDEKEPCPLFLLFSVNASSQFCGVAEMVGPVDFEKSVDYWQQDKWSGQFPVKWHIIKDVPNSQFRHIILENNDNKPVTNSRDTQEVKLEQGIEMLKIFKNYDADTSILDDFGFYEEREKIIQDRKARRQPS
	AT1G27960	ECT9	SKMISYDRVDRFCQQELLSQFRDAKFFVIKSYSEDNVHKSIKHCVWASTKNGNKKLDAAYREAKKKDVACPVFLLFSVNASSQFCGVAEMVGPVDFNTSVEYWQQDRWSGHFPVQWLIVKDVPNSLFRHIIIESNDNKPVTNSRDTQEVGLEKGIEMLDIFISCEMRSSILDDFNFYEERQIAIQDRKARQRAV
	AT5G58190	ECT10	DSSTAGPNPSLYNHPEFVTDYKNAKFFIVKSFSEDNVHRSIKYNVWASTPHGNKKLDTAYRDAEKMGGKCPIFLFFSVNASGQFCGVSEMVGPVDFEKDAGYWQQDRWSGQFPVKWHIVKDIPNNRFCHILLQNNDNKPVTHSRDSQEVKLRQGIEMLRIFKEYEAHTSILDDFGYYDELEGQKVGEDGTRKKA
	AT3G17330	ECT6	AEGNIVINPDRYNKEDFSIEYSDARFFVIKSYSEDDVHKSIKYGVWSSTLNGNKKLQSVYEDAQRIATEKSRECPIFLFFSVNSSGLFCGVAEMTGPVSFDRDMDFWQQDKWSGSFPVKWHIIKDVPNSYFRHIILHNNENKPVTNSRDTQEIILKQGLEVLKLFKHHAEKTSLLDDFMYYEDRQRLMQEERARLPFR
	AT1g48110	ECT7	AEGNIVINPSQYNKEDLRIDYSNAKFFVIKSYSEDDVHKSIKYNVWSSTLHGNKKLQSAYEDAQRIATEKSCECPIFLFFSVNASGLFCGMAEMTGPVSFDKDMDFWQQDKWSGSFPVKWHIIKDVPNSYFRHIILQNNENKPVTNSRDTQEIMLKQGLEVLKIFKDHMERTSLLDDFVYYESRQRVMQDERTRLPYR
	AT1G79270	ECT8	NGVGSVIRRDQYNLPSFQTKYEEAIFFVIKSYSEDDIHKSIKYNVWSSTLNGNKKLDSAYQESQKKAADKSGKCPVFLFFSVNASGQFCGVAEMIGRVDYEKSMEFWQQDKWTGYFPVKWHIIKDVPNPQLRHIILENNENKPVTNSRDTQEVRLPQGNEVLNIFKNYAAKTSILDDFDFYENREKVMVQKKLRFPPV
	AT1G09810	ECT11	NSFALALRREMYNLPDFQTDYEDAKFFVIKSYSEDDVHKSIKYSVWSSTINGNKKLDAAF

			RDAETKTLEDGKKRPIFLFFSVNASRQFVGLAEMVGYVDFNKDLDFWQVDKWSGFFPVEWHVVKDIPNWELRHIILDNNEDKPVTHTRDTHEIKLKEGLQMLSIFKKYSAVTFLLDDMDFYEEREKSLRAKKEHKPAT
	AT1G30460	CPSF30	NRTSHPLPQGVNRYFVVKSNNRENFELSVQQGVWATQRSNEAKLNEAFDSVENVILIFSVNRTRHFQGCAKMTSRIGGYIGGGNWKHEHGTAQYGRNFSVKWLKLCELSFHKTRNLRNPYNENLPVKISRDCQELEPSVGEQLASLLYLEPDSELMAISIAAEAKREEEK
	AT4G11970	ECT12	KANKNSKPGYRTRYFIIKSLNYDNIQVSVEKGIWATQVMNEPILEGAFHKSGRVILIFSVNMSGFFQGYAEMLSPVGWRRDQIWSQGGGKNNPWGRSFKVKWLRLSELPFQKTLHLKNPLNDYKPVKISRDCQELPEDIGEALCELLDANSCDDGLLNSSSRDDYSTKR
Eucalyptus grandis	Eucgr.B03590.1	EgDF1A	NKVSVIPDREQYNKADFPEDYEDAKFFIIKSYSEDDVHKSIKYSVWASTPNGNKKLDAAYREAQEKPGSSPVFLFFSVNTSGQFVGLAEMVGPVDFHKSVEYWQQDKWNGCFPVKWHIVKDVPNSMLKHITLENNENKPVTNSRDTQEVKLVQGLKMIKIFKEHPVKTCLFDDFEFYENRQKAIQEKKARQQYQ
	Eucgr.B03591.1	EgDF2A	NRSSVVPDCTQYNKVDFPEDYNDAKFFIIKSYSEDDVHKSIKYNVWASTPNGNKKLDAAYREAQEKAAGCPIFLFFSVNTSGQFVGLAEMVGPVDFHKNVEYWQQDKWMGCFPVKWHIVKDVPNNLLKHITLENNENKPVTNSRDTQEVKLPQGLKMIKIFQTHLVKTCLLDDFDFYENRQRAIQEKKARQLQF
	Eucgr.J03195.1	EgDF1B	SASITKIPHGSYNHIDFITEYDDAKFFVIKSYSEDNVHKSIKYGVWASTPNGNRKLDAAYREAKEKPDTCPVFLFFSVNASAHFCGVAEMVGPVDFDRSFDYWQQDRWSGQFPVKWHVIKDVPNSQLRHIVLENNDNKPVTNSRDTQEVKLEQGIEMLNIFKNYETDTSILDDFEFYEEREKVIQEGKARQQAS
	Eucgr.H04096.1	EgDF1C	AQGIIIIDTDQYNKDDFCVDYEDAKFFVIKSYSEDDVHKSIKYNVWSSTPHGNKKLQSAYEDAQKIAAEKTTNCPIFLFFSVNASGQFCGVAEMVGQVDFHKNMDFWQQDKWTGSFPVKWHIIKDVQNTAFRHIILENNENKPVTNSRDTQEIMQKQGLEMLKIFKNNGLKTSLLDDFTYYEGRQKLMQEERARMYVS
	Eucgr.E02496.1	EgDC1A	GRGPTPLPPGVSRYFIVKSCNRENLELSVQQGVWATQRSNEAKLNEAFDSAANVILIFSVNRTRHFQGCAKMTSRIGGAASGGNWKYAHGTAHYGRNFSVKWLKLCELSFQKTRHLRNPYNENLPVKISRDCQELEPSVGEQLASLLYLEPDSELMPISLAAESKREEEK
	Eucgr.D01949.1	EgDC1B	SIANRKGRWYSIRYFIIKSLNHHNLQLSIEKGIWATQVMNEPILEEAFHNSGKVILIFSVNMSGFFQGYAQMMSPIGWRRDKVWSQGNGRNNPWGRSFKVKWLRLYDLPFQKTLHLKNLLNDNKPVKISRDCQELPQDVGEALCELLDCDADVDGETRRDSAPLEK
Populus Trichocarpa	Potri.003G222700.1	PtDF1A	DKTTVVPDREQYNKADFPEEYDNAKFFIIKSYSEDDVHKCIKYNVWASTPNGNKKLDAAYQEAEQKSGGCPVFLLFSVNTSGQFVGLAEMTGRVDFDKSVEYWQQDKWTGYFPVKWHIVKDVPNSFLKHITLENNENKPVTNSRDTQEVKLEQGLKLIKIFKDHSSKTCILDDFVFYEDREKMIQEKKAKQQQL
	Potri.001G002000.1	PtDF2A	DKTTEVPDREQYNKADFPVEYVDAKFFIIKSYSEDDVHKCIKYNVWASTPNGNKKLDAAYQEAGQKSGGCPVFLLFSVNTSGQFVGLAEMTGRVDFDKSVEYWQQDKWTGYFPVKWHFVKDVPNSLLKHITLENNENKPVTNSRDTQEVKLEQGLEMIKIFKEHSSKTCILDDFGFYEDREKMIQEKKAKQQQL
	Potri.019G034300.1	PtDF3A	ENLLQIPDREQYNKEDFPEEYSDAKFFVIKSFSEDDVHKSIKYSVWTSTPNGNKKLDAAYKQGKENPGDCPVFLLFSVNTSGQFVGLAEMVGPVDFNKTVEYWQQDKWTGCFPLKWHIIKDVPNGCLRHITLENNENKPVTNSRDTQEVIFEKGVQILKIFKDHKGKTSILDDFSFYAGRERIMQEKRAKHNIH
	Potri.014	PtDF1B	SKPSAKIHDESYNQSDFVTEYKDAKFFIIKSYSEDNVHKSIKYGVWASTPNGNRKLDTAY

G001000.1		HEAKDKQDPCPVFLLFSVNASAQFCGVAEMVGPVDFDKSVDYWQQDKWSGQFPVKW<br>HIIKDVPNSQFRHIVLENNDNKPVTNSRDTQEVKLEQGVEMLNIFKNYETDTSILDDFDF<br>YEDRQKAMQDRKARQQAS
Potri.007 G002800.1	PtDF2B	SKPSAKIHDESYNQPDFVIEYKDAKFFIIKSYSEDNVHKSIKYGVWASTPNGNRKLDTTYR<br>EAKEKQDPCPVFLLFSVNASAQFCGVAEMTGPVDFDKSVDYWQQDKWSGQFPVKWHI<br>IKDVPNSQFRHIVLENNDNKPVTNSRDTQEVKLEQGIEMLNIFKNYETDMSIIDDFDFYE<br>DRQKAMQERKARQQAS
Potri.006 G079900.1	PtDF3B	NSASSGIQLDLYNQLDFVTDYKDAKFFIIKSFSEDNVHKSIKYSVWASTPHGNKKIDAAY<br>REAKEKEGNCPVFLLFSVNASGQFCGVAEMVGPVDFEKDAEYWQQDRWNGQFPVQW<br>HIVKDVPNSRFRHILLENNDNKPATHSRDSQEVKLEQGIEMLKIFKDHDAPTSILDDFDF<br>YDQCERALKERKAKQQPS
Potri.018 G149800.1	PtDF4B	NSASSGVQLDLYNRPVFVTDYKNAKFFIIKSFSEDNVHKSIKYSIWASTPHGNKKIDAAYR<br>EAKEKEGNCPVFLLFSVNASGQFCGVAEMVGPVDFEKDADYWQQDRWNGQFPVQWH<br>IIKDVPNSRFRHILLENNDHKPVTHSRDSQEVKLEQGIEMLKIFKDYDAPTSIIDDFGFYD<br>QCERALKERKAIQQPS
Potri.001 G056100.1	PtDF5B	QKAVADVHSESHNQVDFATDYKDAKFFVIKSYSEDNVHKSIKYGVWASTPNGNKKLD<br>AAYREAKENHGTCPIFLLFSVNASAQFCGVAEMVGPVDFDKNVDFWQQDKWSGQFPV<br>KWHIIKDVPNSQFRHIVLENNDNKPVTNSRDTQEVELEQGAEMLGIFKNYESHSSILDDF<br>QFYEERQKVMQVRKSRPQAS
Potri.010 G152300.1	PtDF1C	GLGNIVIQTDQYNKDDFSTDYLDAKFFVIKSYSEDDVHKSIKYNVWSSTPHGNKKLQTAF<br>EDAQKLAVGRPRGCPIFLFFSVNASGQFCGVAEMIGPVDLHRDMDFWQQDKWSGSFLV<br>KWHIIKDIPNSSFRHIILENNENKPVTNSRDTQEIMYKQGLEMLKTFKNHPLRTSILDDFM<br>YYENRQKIMQDEKARLMFK
Potri.008 G100200.1	PtDF2C	ELGNIVIQTDQYNKDDFSTDYADAKFFVIKSYSEDDVHKSIKYNVWSSTPHGNKKLHSAF<br>EYAQKLDLGRPRGCPIFLFFSVNASGQFCGVAEMVGPVDFNRDMDFWQQDKWSGSFLV<br>KWHIIKDIPNSSFRHIILENNENKPVTNSRDTQEIMYKQGLEMLKMFKNHPLKTSILDDF<br>VYYENRQKIMQEEKARLMFK
Potri.010 G175500.1	PtDF3C	NSITSVISRDQYNLPDFPTNYDHAFFFVIKSYSEDDIHKSIKYNVWASTPNGNKRLNSAYL<br>DSQQKIAQIGCSCPVFLFFSVNASGQFCGVAEMTGRVDFNKNMDFWQQEKWNGYFPV<br>KWHIIKDIPNPQLRHIILENNENKPVTNSRDTQEVKFPQGIEILNIFKNYVSKTSILDDFDF<br>YESRQKVMHERRPRSLIS
Potri.008 G080800.1	PtDF4C	NSIASVISRDQYNLPDFPTKYNHAFFFVIKSYSEDDIHKSIKYNVWASTPNGNKRLDSAYQ<br>DAQQKIAEKGNSCPVFLFFSVNASGQFCGVAEMVGRVDFNKNMDFWQQDKWNGYFP<br>VKWHIIKDVPNPQLRHIILENNENKPVINSRDTQEVKFPQGIEILNIFKNYVTKTSILDDFD<br>FYESRQKVMQEKRPRPFIP
Potri.004 G223800.1	PtDF5C	DMLGFAMHKEQYNLPDFEIEYSNAKFFVIKSYNEDDIHKSIKYDVWASTPNGNKKLDA<br>AFHNAEEVSSETGTKCPIFLFFSVNGSGQFVGLAEMVGQVDFNKDMDFWQIDKWNGFF<br>PVKWHVIKDIPNGQLRHIVLENNDGHSVTFSRDTQEIGLEKGLEMLNIFKSYSAKTSMLD<br>DFNFYENREKSLNTKKSNKPAT
Potri.003 G008400.1	PtDF6C	DELGIAMRKEQYNLPDFETEYANAKFFVIKSYSEDDIHKSIKYDVWASTPNGNKKLDAA<br>FHNAEEVSSDTGYKCPIFLFFSVNGSGQFVGFAEMVGQVDFNKDMDFWQIDKWNGFFP<br>VKWHVVKDIPNGHLRHIVLENNDGHSVTFSRDTQEIVLKQGLEMLNIFKSYSAKTSLLD<br>DFNFYEKREKSLNTKKGNKPAT
Potri.001	PtDC1	SSFFSCVSPSQFVYFIVKSCNRENLELSVQQGVWATQRSNEIKLNEALDSADNVILIFSVNR

	G357800.1	A	TRHFQGCAKMASKIGASVGGGNWKYAHGTAHYGRNFSVKWLKLCELSFHKTRHLRNP<br>FNENLPVKISRDCQELEPSIGEQLASLLYLEPDSELMAVSLAAEAKREEEK
	Potri.011<br>G089800.1	PtDC2<br>A	VLYVVVFWLRVKLYFIVKSCNLENLELSVQQGVWATQRSNEPKLNEAFDSAENVILIFSV<br>NRTRHFQGCAKMTSKIGASVGGGNWKYAHGTAHYGRNFSVKWLKLCELSFHKTRHLR<br>NPFNENLPVKISRDCQELEPSIGEQLASLLYLEPDSDLMAISVAAEAKREEEK
	Potri.001<br>G113500.1	PtDC1B	SSINKKRKLCNTRYFIIKSLNQHNIQLSIENGIWATQVRNEPILEEAFHNSGRVILIYSVNM<br>SGFFQGYAQMISSVGWRHDNLWSEGSGKSNPWGRSFKVKWLRLNDLPFQKTLHLKNPL<br>NDYKPVKISRDCQELPEDIGEALCELIDGERDTDGMVKSFPRDDLPMKR
	orange1.1<br>g005158m	CsiDF1A	DKISLSPDRDEYNKADFPEEYTDAKFFVIKSYSEDDVHKSIKYSVWASTPNGNKKLDAAY<br>QEAQQKSRSCPVFLLFSVNTSGQFVGLAEMAGPVDFNKNVEYWQQDKWTGCFPVKW<br>HIVKDVPNSLLKHITLENNENKPVTNSRDTQEIKLEQGLKLIKIFKDHPSKTCILDDFGFY<br>ETRQKTIQEKKAKQQQF
	orange1.1<br>g010141m	CsiDF2A	DNLPLIPDKEKYSGEDFPESYSDAKFFIIKSYSEDDVHKSVKYNMWTSTPNGNKKLDAAY<br>REAKEKSSDCPVFLLFSVNASGQFVGVAEMVGPVDFDKTVEYWQQDKWVGCFPLKWLI<br>IKDVPNSSLRHITLENNENKPVTNSRDTQEVNFEIGIQILKIFKSHSSKRCILDDFGFYEARE<br>RIMQQKKAKQHQL
	orange1.1<br>g005934m	CsiDF1B	SKSVVKILDESYNQPDFVTDYKDAKFFIIKSYSEDNVHKSIKYGVWASTPNGNKKLDAAY<br>CEAKEKQDPCPVFLFFSVNASAQFCGVAEMIGPVDFEKSVDYWQQDKWSGQFPVKWHI<br>IKDVPNSQFRHIVLENNDNKPVTNSRDTQEVKLEQGIEMLNIFKNYVTDMSILDDFDFYE<br>DRQKAMQERKARQQAS
	orange1.1<br>g038459m	CsiDF2B	NSSGSGINFELYNQFDFPTDYENAKFFVIKSFSEDNVHKSIKYGVWASTPHGNKKLDAAY<br>HEAKETDNRCPIFLLFSVNASGQFCGVAEMVGPVDFENSADYWQQDRWSGQFPVKWLI<br>IKDVPNSRFRHLLLENNDNKPVTHSRDSQEVKLEQGVEMLRIFKEHDARTSILDDFDFYD<br>ERERSLKERRAKQQVS
Citrus<br>sinenss	orange1.1<br>g006798m	CsiDF3B	NGTLDGILKGSYNQLDFVTNYKVAKFFIIKSYSEDNVHKSIKYGLWASTPNGNKKLDAA<br>YREAKEKHGTCPIFLLFSVNASAQFCGVAEMVGPVDFDKSVDYWLQDKWSGQFPVKW<br>HIIKDVPNSQFRHIILEKNDNKPVTNSRDTQEVELEQGIEMLNIFKNYESYSSILDDFHFYE<br>QRQKAMQERKAGQQAN
	orange1.1<br>g005441m	CsiDF1C	VEGNIIICTEHYNKEDFQVDYVDAKFFVIKSYSEDDVHKSIKYGVWSSTSHGNKKLQSAY<br>EDAQKLAAGKPGGCPIFLFFSVNASSQFCGVAEMIGPVDFYKDMDFWQQDKWSGSFPV<br>KWHIIKDVPNTSLRHIILRNNENKPVTNSRDTQEIMHTEGLEMLKIFKNHPRDTSLFDDF<br>MYYEKRQKFMQEERARLVFK
	orange1.1<br>g008255m	CsiDF2C	EVLSPTVSRDQYNLPDFQVEYEKAKFYVIKSYSEDDIHKCIKYDVWSSTPNGNKKLDATF<br>NEAEAKADETGTRCPIFLFFSVNGSGQFVGLAEMMGKVDFNKDMDFWQLDKWNGFFP<br>VKWHVIKDVPNTLLRHITLENNENKPVTHSRDTQEIGLKQGLEMLKIFKSYSAKTSLLDD<br>FNFYENKERSFHGKKSSKPAT
	orange1.1<br>g048663m	CsiDF3C	DSISSLIWKDQYNLPDFRIKYDHALFFVIKSYSEDDIHKSIKYSVWSSTPNGNKKLDAAYE<br>DAQSRIAEKGSKCPVFLFFSVNASGQFCGVAEMIGRVDFNKNMDFWQQDKWNGYFPV<br>KWHIIKDVPNPQLRHIILENNDKKPVTNSRDTQEVKFPQGIEILNIFKNYPSKTSILDDFDF<br>YESRQKVMQEKKVRLSVS
	orange1.1<br>g005338m	CsiDC1A	NRNATPLPQGISRYFIVKSCNRENLELSVQQGVWATQRSNEAKLNEAFDSAENVILIFSV<br>NRTRHFQGCAKMTSKIGGSVGGGNWKYAHGTAHYGRNFSVKWLKLCELSFHKTRHLR<br>NPYNENLPVKISRDCQELEPSIGEQLAALLYLEPDSELMAISVAAEAKREEEK
	orange1.1	CsiDC1B	STDNNKGKLYNTRYFIIKSLNHQNIQLSIEKEIWATQVMNEPILEEAFHNSGKVILIFSVN

	g015749m		MSGFFQGYAQMMSSVGWRRDNVWSQGNGKNNPWGRSFKVKWLRLNTLPFQKTLHLKNPLNDYKPVKISRDCQELPQDIGEALCHLLDGKDDVDGIQTSFHRDDLPAKR
Camellia chekiangoleosa	Cch01T002587.1	CchYTH1	NAKFFVIKSYSEDDVHKGIKYNVWSSTPNGNKKLQSAYEDAQRIASGEPRGCAIFLFFSVSDIPLILSFALKYYIPQVNASGQFCGVAEMTGPVDFHKDMDFWQQDKWSGSFPVKWHIIKDVPNPNFRHIILENNENKPVTNSRDTQEIRCRQGIEMLKIFKLYTSK
	Cch02T002736.1	CchYTH2	NAKFFVIKSYSEDDVHKGIKYNVWSSTPNGNKKLRSAYEDAQRIAFGEPRGCPIFLFFSVRDIPLILSFALKYYIPQVNASGQFCGVAEMTGPVDFHKDMDFWQQDKWSGSFPVKWHIIKDVPNPNFRHIILENNEN KPVTNSRDTQEMFFSTHYAGDNLTIKLRD
	Cch02T003694.1	CchYTH3	DAKFFIIKSYSEDNVHKSIKYGIWASTPNGNRKLDAAYREAKEKNTCPVFLFFSVNASAQFCGVAEMVGPVDFDKSVDYWQQDKWSGQFPVKWHIIRDVPNSQFRHIVLENNDNKPVTNSRDTQEAKLEQGIEMLKIFNNYES
	Cch03T002158.1	CchYTH4	NAKFFIIKSFSEDNVHKSIKYSVWASTPLGNRKLDAAYHEVKDANGHCPVFLLFSVNASGQFCGVAEMIGPVDFEHDVDYWQQDRWSGQFPVRWHIIKDVPNNQFRHILLENNDNKPVTHSRDSQEVKLEQGIEILKIFKDYEA
	Cch05T000069.1	CchYTH5	NAKFFVIKSYSEDDVHKSIKYNVWSSTPNGNKKLQSAYEDARRIAAGEPRGCPVFLFFSVNASGQFCGVAEMTGPVDFYKDMDFWQQDKWSGSFPVKWHIIKDVPNPNFRHIILENNENKPVTNSRDTQEVCFVFIPDTLFTC
	Cch07T000910.1	CchYTH6	NAKFYVIKSYSEDDIHKCIKYDVWSSTPNGNKKLDAAFHDAEAKACETGTKCPIFLFFSVNGSGQFIGVAEMIGPVDFNKDMDFWQLDKWNGFFPVKWHIIKDTPNTQLRHIILENNDNRSVTYSRDTQEIGFKQGIEMLNIFNSYSE
	Cch08T001554.1	CchYTH7	ISRYFIVKSCNRENLELSVEQGVWATQRSNEAKLNEAFDSVENVILIFSVNRTRHFQGCAKMTSKIGGSVGGGNWKYAHGSAHYGRNFSVKWLKLCELSFHKTRLLRNPYNENLPVKISRDCQELEPSVGEQLASLLYLEPD
	Cch09T004551.1	CchYTH8	NAKFFVIKSYSEDDVHKGIKYNVWSSTPNGNKKLQSAYEDAQRIASGEPRGCAIFLFFSVSDIPLILSFALKYYIPQVNASGQFCGVAEMTGPVDFHKDMDFWQQDKWSGSFPVKWHIIKDVPNPNFRHIILENNENKPVTNSRDTQEIRCRQGIEMLKIFKLYTS
	Cch12T001174.1	CchYTH9	DAKFFIIKSYSEDDVHKSIKYNVWASTPNGNKKLDAAYQEAQQKSGGCPVFLFFSVNTSGQFVGVAEMTGPVDFHKNFEYWQQDKWNGCFPVNWHIVKDVSNSLLKHITLENNENKPVTNSRDTQEVKLEQGLQLLKIFKDHSSK
	Cch13T001809.1	CchYTH10	GTRYFIIKSLTHQNIQLSIEKGIWATQVMNEPILEEAFHNSSKVILIFSVNMSGFFQGYAQMMSSVGWRRDNVWSQGSGGKNPWGRSFKVKWLRLHDLPFQKTLHLKNPLNDYKPVKISRDCQELPQDIGEALCELLDGKDD
Liriodendron chinense	Lchi30795	LcYTHDF1	AKFFIIKSYSEDDIHKSIKYGVWSSTEHGNKKLDFCYQEAREKSGGCPVFLFFSVNSSGQFVGLAEMVGPVDFNKNVEHWQQDKWNGCFPVKWHIVKDVPNSTLKHITLENNDNKPVTNSRDTQEVKLDQGLEVLQIF
	Lchi30011	LcYTHDF2	AKFFVIKSYSEDDIHKSIKYNVWASTVGGNQKLNAAYQEAQEKLGGCPVFLFFSVNTSGQFVGVAEMEGPVDFNKSVEYWQQDKWTGCFPVKWHIVKDVPNSLLKHIKLENNEDKPVTNSRDTQEVKFDQGLEILKIF
	Lchi10804	LcYTHDF3	AKFFIIKSYSEDNVHKSIKYSVWASTSGGNRRLDAAYRESKEKEGACPVFLLFSVNASAQFCGVAEMVGLVDFDKSVDYWQQDKWTGQFPVKWHVIKDVPNSQFRHITLENNENKPVTNSRDTQEVGLEQGLEMLNIF
	Lchi05236	LcYTHDF4	AKFFIIKSYSEDNVHKSIKYGVWASTTSGNRKLDAAYHESKEKEGACPVFLLFSVNASAQFCGVAEMMGPVDFDKSVDYWQQDKWSGQFPVKWHIIKDVPNSQFRHIILENNDNKPVTNSRDTQEVGLEQGIEMLNIF

	Lchi06362	LcYTHDF5	AKFFVIKSYSEDDVHKSIKYSVWSSTPSGNKRLDSAYEDAQRISGGRPGNCPVFLFFSVNASGQFCGVAEMTGPVDFQKDMDFWQQDKWNGSFPVKWHIIKDVPNANFRHIILENNENKPVTNSRDTQEIRYMQGIDMLNIF
	Lchi04058	LcYTHDF6	AFFYVIKSYSEDDVHKSIKYNVWASTPNGNKRLDSAYQDAQERTGEKGSKCPVFLFFSVNASGQFCGVAEMTGHVDFNKNMDFWQQDKWNGFFPVKWHIIKDVPNPQFRHVILENNENKPVTNSRDTQEVKFPQGIEMLNIF
	Lchi07049	LcYTHDF7	HALFYVIKSYSEDDVHKSIKYNVWASTQNGNKRLDDSYQAAQERMEEKGCKCPIFLFFSVNASGQFCGVAEMVGRVDFNKSMEFWQQDKWNGSFPVKWHIIKDIPNPQFRHIILENNENKPVTNSRDTQEVRFHQGIEMLNIF
	Lchi01573	LcYTHDF8	AAVLTATKPNVSAPSPSASLKLKGMNSTKTKGTSDTKASAPPSDSKSRASIASSNFLNSSFHSQPMKPANKVNASGQFVGLAEMIGQVDFKKNMDFWQFNGKWGGFFPLKWHMIKDIPNNQFLHIILENNDYNSVTFSKDTQEIGFPQGLEMLKIF
	Lchi11130	LcDC1	RYFIVKSCNRENLELSVQQGVWATQRSNEAKLNEAFDSSENVILIFSINRTRHFQGCAKMTSKIGGFVGGGNWKYAHGTAHYGRNFSVKWLKLCELSFHKTRHLRNPYNENLPVKISRDCQELEPSIGEQLASLL
	Bol035532	BoDF1A	ENTCIVPDREQYNKEDFPVDYADAMFFIIKSYSEDDVHKSIKYNVWASTPNGNKKLAAAYQEAQQKPGGCPIFLFFSVNASGQFVGLAEMTGPVDFNTNVDCWQQDKWTGSFPLKWHIVKDVPNSLLKHITLENNENKPVTNSRDTQEVKLEQGLKIVKIFKEHTSKTCILDDFSFYEVRQKTILEKKAKQTQK
	Bol040382	BoDF2A	ENTCIVPDREQYNKEDFPVDYENAMFFVIKSYSEDDVHKSIKYNVWASTPNGNKKLAAAYQEAQQKPGGCPIFLFFSVNASGQFVGLAEMTGPVDFNTNVEYWQQDKWTGSFPLKWHIVKDVPNSLLKHITLENNENKPVTNSRDTQEVKLEQGLKIVKIFKEHTSKTCILDDFSFYEVRQKTILEKKAKQQQT
	Bol015814	BoDF3A	ENTCIVPDREQYNKEDFPVDYENAMFFVIKSYSEDDVHKSIKYNVWASTPNGNKKLAAAYQEAQQKPSGCPIFLFFSVNASGQFVGLAEMTGPVDFDTNVEYWQQDKWTGSFPLKWHIVKDVPNSLLKHITLENNENKPVTNSRDTQEVKLEQGLKIVKIFKEHTSKTCILDDFSFYEVSEEKTATDEKKESSTA
Brassia oleraca	Bol012589	BoDF4A	PEDVSLPDSKEYNKEDFSDSYYSNAKLFVIKSYSEDDVHKSIKYNVWSSTPNGNKKLNAAYNEAKDKSCPVFLIFSVNTSGQFVGLAEMVGPVDFNQTVEYWQQDKWVGCFPVKWHIVKDIPNSSLRHITLENNENKPVTNSRDTQEVKVEQGVKVIKIFKEHESKTCILDDFVFYESREKIIKEGKKKHQEY
	Bol035536	BoDF5A	VTEAVAPDREQYNKEDFPVDYANAKFFVIKSYGEDDVHKSIKYNVWASTLRGNKKLDAAYEAAQQKPRACPVFLFFSVNTSRHFVGLSEMTGRVEFDKNVDYWQQDKWKGSFPLRWHIVKDVPNSLLKHIILDNRKRVTRSRDTEEVMLEQGLNILKIFKEHTSKTCILDDFSFYEVREKTILEKKAK
	Bol010247	BoDF1B	NKGSTKEHEDSNNNSNDFVTDYNDAKLFIIKSYSEDNVHKSIKYNVWASTANGNKKLDAAYREAKEEKEPCPVFLLFSVNASSQFCGVAEMIGPVDFEKSVDYWQQDKWSGQFPVKWHIIKDVPNSQFRHIILENNDNKPVTNSRDTQEVKLEQGIEMLKIFKSYDAETSILDDFEFYEEREKIIQDRKARRQPS
	Bol015779	BoDF2B	NKDSPKEEANNNNNPEFVTDYSDAKLFIIKSYSEDNVHKSIKYNVWASTPNGNKKLDAAYREAKEEKEACPVFLLFSVNASSQFCGVAEMIGPVDFEKSVDYWQQDKWNGQFPVKWHIIKDVPNSQFRHIILENNDNKPVTNSRDTQEGIEMLKIFKNYDAETSILDDFGFYEEREKIIQERKARRLPN
	Bol008438	BoDF3B	SRPNAKSKMITYDQQDLLSKFRDAKFFVIKSYSEDNVYKSMKYCVWASTKNGNKKLDA

		AYREAKTKQVACPVFLLFSVNASAQFCGVAEMVGPVDFETSVEYWQQDRWSGHFPVK WVIVKDVPNSLFRHIIIEDNDNKPVTNSRDTQEVGVEQGIEMINIFNSCEMKSSILDDFSFY EERQRIQDRKARQRAL
Bol015553	BoDF4B	GSSTAGPNPSLYNSPEFVTDYKNAKFFIVKSFSEDNVHRSIKYNVWASTPHGNKKLDTAY RDAEKMGGKCPIFLFFSVNASGQFCGVSEMVGPVDFEKDAGYWQQDRWSGQFPVKWH ILKDVPNNRFSHILLQNNDNKPVTHSRDSQEVKLRQGIEMLRIFKEYEAHTSILDDFSYYD ERERQKVGEDGGRKED
Bol025994	BoDF5B	DSSTAGSNPSLYNHPEFATDYKNARFFIVKSFSEDNVHRSIKYNVWASTPHGSKKLDTAY RDAEKMGGKCPIFLFFSVNASGQFCGVAEMVGPVDFEKDAAYWQQGKWNGQFPVKW HIVKDVPNNRFSHILLQNNDNKPVTHSRDSQEVKLRQGIEMLRISKEYEAHTSILDDFSY YDERESEKVGEDGVRKEE
Bol045155	BoDF1C	AEGNIVIDPNLYNKEDLRIDYTNAKFFVIKSYSEDDVHKSIKYNVWSSTLHGNKKLQSAY EDAQRIATEKSCECPIFLFFSVNASGLFCGMAEMTGPVSFEKDMDFWQQDKWSGSFPVK WHIIKDVPNSYFRHIILHNNENKPVTNSRDTQEIMMKQGLEVLKIFKGHAERTSLLDDFA YYENRQRVMHDERNRLPYR
Bol036797	BoDF2C	SSFVLDLRREMFNLPDFQTDYEDAKFFVIKSYSEDDVHKSIKYSVWSSTVNGNKKLDAAY RDAEAKTLVDGKKRPIFLFFSVNASRQFVGLAEMVGYVDMNKDLDFWQVDKWCGFFP VEWHVVKDVPNWELCHIVLDNNEGKAVTHTRDTQEIKLREGLQMLSIFKKFSAVTSLL DDMDFYEEREKSLRLKKEHKPAT
Bol016106	BoDC1A	DQTSHPLPRGVNRYFVVKSNNSENFELSVQQGVWATQRSNEAKLNEAFDIVDNVILIFS VNRTRHFQGCAKMTSRIGGYIGGGNWKNEHGTQQYGGNFSVKWLKLCELSFHKTRNL RNPYNENLPVKISRDCQELEPSVGEELASLLYLEPDSKLMAISIAAEAKREEEK
Bol044954	BoDC1B	GSKNKSKPGYQTRYFIIKSLNYDNIQLSVERGIWATQVMNEPILEGAFHNSGRVVLIFSVN MSGFFQGYAEMLSPVGWRRDHIWSQGGGKNNPWGRSFKVKWLRLTELPFQKTLHLKN PLNDYKPVKISRDCQELPGDIGEALCELLDAHSCDDGLLNRDDYSTKR

* All accession numbers relate to the JGI Phytozome, Metazome or MycoCosm databases

** In cases when a name was already attributed to a protein, this name was kept with the new name, refering to the species of origin and to the phylogenetic classification of the protein, added in parenthesis

*** For these species, only the core YTH motif are indicated

Table S2. Primer sequences.

**PrimerName**	**Primer sequences(5'-3')**
MgYTH5-F	ATGAATAACAACGAAAACTTGGA
MgYTH5-R	AAGCCTTTTTTTTTCTTGCATTGC
MBP-MgYTH5-F	CTTCCAATCCCATATGGCCATGAATAACAACGAAAACTTGGA
MBP-MgYTH5-R	atggtgatggtgatggtgAAGCCTTTTTTTTTCTTGCATTGC
MBP-F	CCGCTTTCTGGTATGCCG
MBP-R	GATCCGGTACCAAGCTTGTTGGA
Q-MgYTH1-F	ACCAAGATTGCACAGCATGG
Q-MgYTH1-R	CCATTCATGGCCATTGGCTT
Q-MgYTH2-F	GAAGCAGCAAGTTCATCAGCC
Q-MgYTH2-R	ACTGGCAACCGAACTTCTGT
Q-MgYTH3-F	TGCAGAGACACCAATTGCACC
Q-MgYTH3-R	GGTGGATTATCTGGTGGAAATGTCC
Q-MgYTH4-F	TGAACAGAACCGTGGTCCTC
Q-MgYTH4-R	TTGGTCCCTACCAGCTACTG
Q-MgYTH5-F	GGGCTAACACTCCTAATGGGAACA
Q-MgYTH5-R	GCCACTAGCATTAACCGAGAAG
Q-MgYTH6-F	GGAAGGATTTGCCATGCCAGAAA
Q-MgYTH6-R	CCCATGGAAGATCATTCCAGGA
Q-MgACT2-F	GACGCTTATGTTGGTGATGAGGC
Q-MgACT2-R	GAGTCATCTTCTCTCTGTTTGCCTTAGG
